# The data quality and applicability of a Danish prehospital electronic health record: A mixed-methods study

**DOI:** 10.1371/journal.pone.0293577

**Published:** 2023-10-26

**Authors:** Jeannett Kjær, Louise Milling, Daniel Wittrock, Lars Bak Nielsen, Søren Mikkelsen

**Affiliations:** 1 Prehospital Research Unit, Department of Anaesthesiology and Intensive Care, Odense University Hospital, Odense, Denmark; 2 Department of Regional Health Research, University of Southern Denmark, Odense, Denmark; 3 Ambulance Syd, Region of Southern Denmark, Odense, Denmark; 4 Responce, Kolding, Denmark; South African Medical Research Council (SAMRC) / Stellenbosch University (SU), SOUTH AFRICA

## Abstract

**Background:**

Without accurate documentation, it can be difficult to assess the quality of care and the impact of quality improvement initiatives. Prehospital lack of documentation of the basic measurements is associated with a twofold risk of mortality. The aim of this study was to investigate data quality in the electronic prehospital patient record (ePPR) system in the Region of Southern Denmark. In addition, we investigated ambulance professionals’ attitudes toward the use of ePPR and identified barriers and facilitators to its use.

**Method:**

We used an explanatory sequential mixed-methods design. Phase one consisted of a retrospective assessment of the data quality of ePPR information, and phase two included semi-structured interviews with ambulance professionals combined with observations. We included patients who were acutely transported to an emergency department by ambulance in the Region of Southern Denmark from 2016 to 2020. Data completeness was calculated for each vital sign using a two-way table of frequency. Vital signs were summarised to calculate data correctness. Interviews and observations were analysed using thematic analysis.

**Results:**

Overall, an improvement in data completeness and correctness was observed from 2016–2020. When stratified by age group, children (<12 years) accounted for the majority of missing vital sign registrations. In the thematic analysis, we identified four themes; ambulance professionals’ attitudes, emergency setting, training and guidelines, and tablet and software.

**Conclusion:**

We found high data quality, but there is room for improvement. The ambulance professionals’ attitudes toward the ePPR, working in an emergency setting, a notion of insufficient training in completing the ePPR, and challenges related to the tablet and software could be barriers to data completeness and correctness. It would be beneficial to include the end-user when developing an ePPR system and to consider that the tablet should be used in emergency situations.

## Introduction

As electronic health records (EHR) become increasingly widespread, the need to assess and understand the use and impact increases [1]. This evidence is important to improve EHR capabilities but also to identify gaps where more targeted investments and evaluations are needed [1]. In a survey of Topaz et al, system usability was the most reported concern with almost one-third of the respondents (31.1%) identifying multiple usability issues [2].

Prehospital documentation can be used to review how patients are being treated and assess how ambulance professionals adhere to common practice guidelines and protocols [3]. Without appropriate documentation of patient care, quality assurance, and improvement mechanisms, it may be difficult to assess the quality of care [3]. A study examining patient safety reports found that poor EHR usability contributed to adverse harmful events [4]. Ambulance professionals’ lack of documentation of the basic measurements of heart rate (HR), systolic blood pressure (sBP), and respiratory rate (RR) at the scene is associated with more than a twofold risk of mortality [5]. Intuitively, this lack of documentation may stem from the ambulance personnel having their hands full when treating patients with a more severe level of illness. However, using a propensity matched model adjusting for the severity of the injury and the physiology parameters measured in the emergency department, the point estimates regarding mortality remained unchanged [5].

It is crucial to include end-users in the process of design, purchase, upgrade, and implementation decisions to improve EHR [2]. Building a deeper understanding of end-user experiences is especially important because of the policymakers’ continued commitment to advance and enhance EHR use [6]. To our knowledge, there are only a few studies examining the satisfaction with EHR use in a pre-hospital setting. Most research has focused on EHR implementation, while empirical information on user perceptions after several years of EHR use is scarce.

### Aim

This mixed-methods study aimed to investigate the data quality in the Prehospital Patient Record in the Region of Southern Denmark concerning data completeness and data correctness.

In addition, we examined the ambulance professionals’ attitudes toward the use of the electronic Prehospital Patient Record (ePPR) and sought to identify the barriers and facilitators in the ambulance professionals’ use of the ePPR in the Region of Southern Denmark.

## Method

### Study design

This study used an explanatory sequential mixed methods design with a quantitative phase followed by a qualitative phase. Phase one consisted of a retrospective data quality assessment of patient record information while phase two involved semi-structured interviews of the emergency medical providers combined with participant observations. The quantitative data were collected first. Ensuingly, seeking to elaborate on these results, the qualitative data were collected and analysed. We used an inductive and hermeneutic phenomenological approach to examine the EHR users’ experiences. Hermeneutic phenomenology is a qualitative research method that allows researchers to study how experiences, traditions, and culture shape ordinary, everyday practices [7].

**Fig 1:**
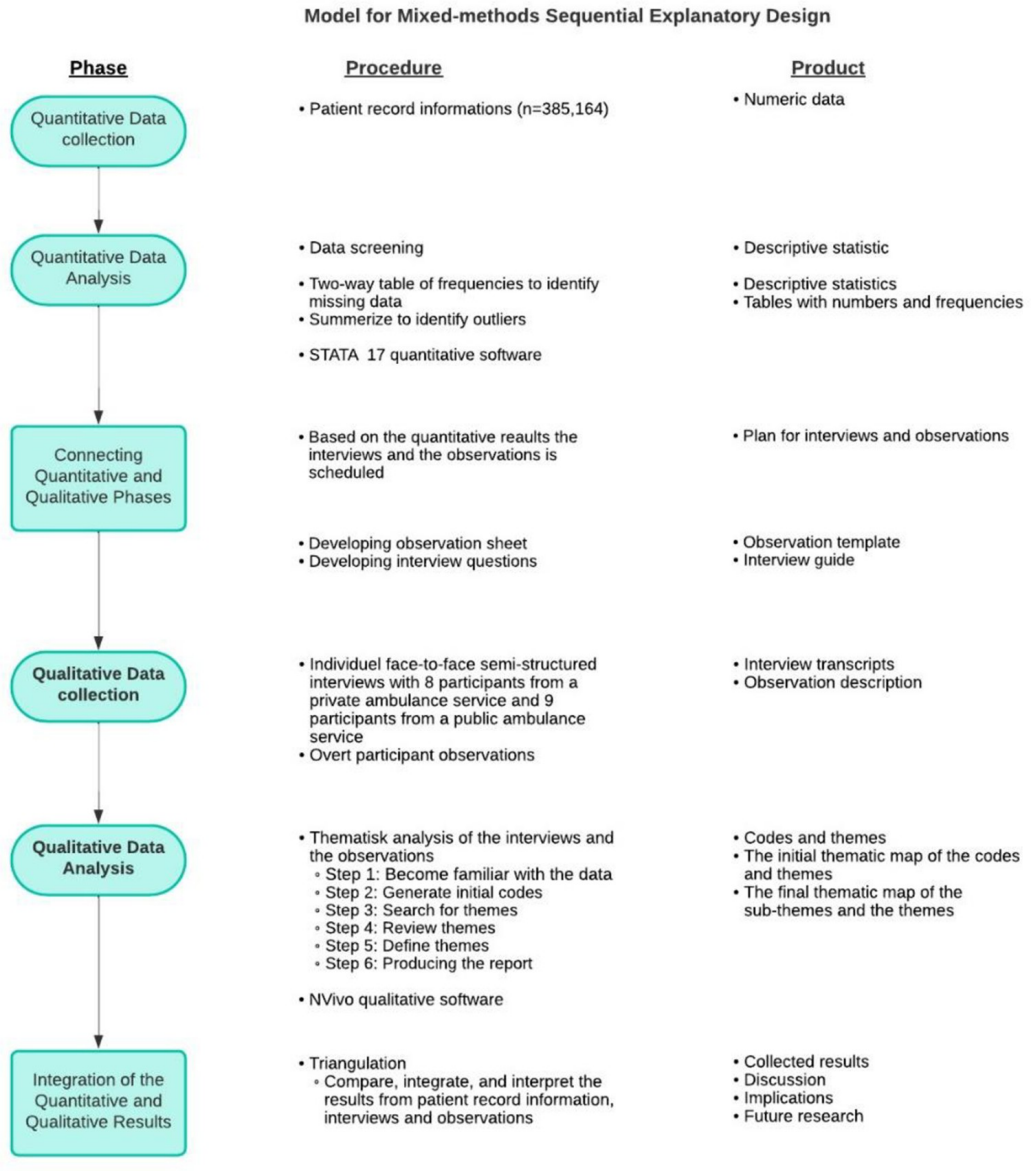
Model for mixed-methods sequential explanatory design. The approach written in bold shows the priority of the methods.

A mixed-method model was developed guided by the ten rules for drawing visual models for mixed-methods design explaining the process of and relations between the quantitative and qualitative phases [8]. Please see Fig 1.

### Study setting

The Danish Emergency Medical Services (EMS) include ambulances, supplemented with rapid response vehicles as first responders, and mobile emergency care units and helicopter emergency medical services (HEMS) manned with prehospital anaesthesiologists [9]. There are two levels of ambulance professionals, emergency medical technicians (EMTs) and paramedics. The ambulances are always manned by at least two ambulance professionals. The prehospital anaesthesiologists can be dispatched via the emergency medical dispatch centre as a supplemental resource [10].

In Denmark, every resident is assigned a personal identification number in The Danish Civil Registration System. It is a unique ten-digit Civil Personal Registration (CPR) number. This number is used to provide a unique identifier in the Danish health care system [11, 12].

Since 2015, all Danish medical emergency vehicles except the HEMS, use the ePPR. The ePPR is installed on a specially designed computer tablet [9]. Ambulance professionals can enter patient data and forward information to hospitals through the EHR [9]. In the ambulances, the ePPRs are wirelessly connected to a server for data synchronisation. However, they can also function without any real-time or direct connection to the server. Therefore, they can withstand losing the connection to the server and log the data when regaining it. A central ePPR in the emergency departments allows the in-hospital healthcare professionals to access ePPR data. The coordinating nurse receives handover information, delegates patients, and keeps an overview in emergency departments [10].

From 2015–2017, the ambulance professionals had access to written educational materials, images and video sequences to instruct them in how to fill in the ePPR. From 2017, the ambulance professionals have received training in filling in the electronic record on training days.

The ambulance personnel has local internal guidelines on how to register vital signs. A monitor in each ambulance transfers non-invasive BP, blood oxygenation saturation (SpO2), and HR wirelessly to the ePPR. When applied, a specialised nasal catheter enables monitoring of end-tidal CO2 and RR [13]. The Glasgow Coma Scale (GCS) scores have to be estimated and entered manually [13].

### Data triangulation

**Fig 2:**
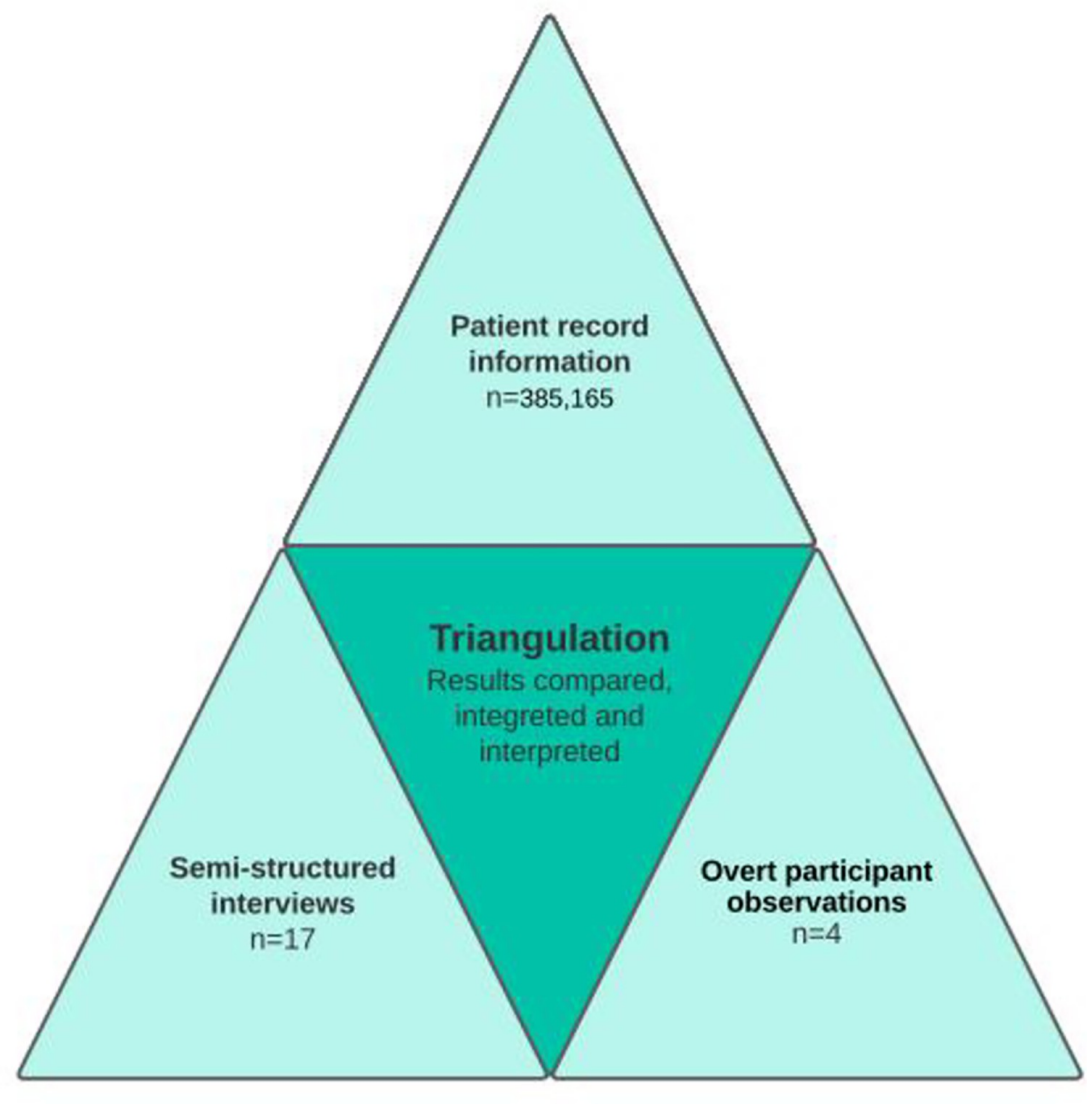
Triangulation.

We used methodological triangulation to create a deeper understanding of the data and to ensure their completeness. Triangulation can be beneficial to explore and explain complex human behaviour using a variety of methods to explain different aspects of a phenomenon of interest [14]. All collected data were analysed individually and then triangulated. Please see Fig 2.

#### First phase: The quantitative analysis

*Data collection*. We included medical records from prehospital patients transported acutely by ambulance to an emergency department in the Region of Southern Denmark from 1 January 2016 to 31 December 2020. Data were extracted from the prehospital medical record system and were made available to the research group as a Microsoft Excel file (Microsoft, Redmond, Virginia, USA).

We assessed the data quality based on two dimensions of data quality identified by Weiskopf and Weng; completeness and correctness [15]. Completeness referred to whether or not a truth about a patient was present in the EHR. Weiskopf and Weng considered EHR data correct when the information they contained was true [15]. We investigated the completeness and correctness of the registered vital signs, the Civil Personal Registration number, and the initial triage level from 1 January 2016 to 31 December 2020. We defined vital signs as; sBP, HR, SpO2, RR, GCS. All vital signs data were extracted from the regional ePPR database.

*Statistics*. We included the last registered measurement of each of the five vital signs for every patient in the quantitative analysis. The vital signs were summarised to calculate data correctness from the minimum and maximum values of each vital sign. Clinically implausible values for sBP were defined as sBP > 300 mmHg [16]. HR was accepted up to 300 beats per minute and RR >100 breaths per minute. The two last vital signs had well-defined cut-points; SpO2 >100% and, the GCS score >15 [16]. For vital signs with incorrect values, we used a one-way table of frequency to create an overview of the number of impossible values.

Data completeness was calculated for each vital sign using a two-way table of frequency to visualise the development of missing data from 2016 to 2020. We stratified the data completeness for each vital sign by age to investigate the differences between adults and children. Furthermore, we calculated the frequency of patients who were missing all five vital signs. The CPR numbers were also investigated for data completeness. The Statistical analyses were performed with STATA version 17 [17].

#### Second phase: The qualitative analysis

*Data collection*. In the qualitative phase, we included ambulance professionals from a public and a private ambulance service provider in the Region of Southern Denmark through convenience sampling. The interviews were conducted during the working hours while the ambulance professionals were available for ambulance runs. Recruitment took place through face-to-face invitations of the attending ambulance professionals.

We developed the interview guide and observation checklist based on the quantitative results from the first phase. The questions were categorised in three themes identified in a systematic review, which investigated perceptions of healthcare professionals about the adoption and use of EHR; perceived benefits, perceived barriers, and perceived influencing factors [18]. We pilot tested the interview guide on two test subjects who were familiar with the ePPR. Based on this pilot interview analysis, we reworded and removed questions. Please see S1 File for the interview guide.

The semi-structured interviews were conducted in March and April 2022 by one interviewer. The interviews took place in familiar surroundings on the ambulance stations. All the interviews were recorded and transcribed verbatim. Sampling continued until saturation was reached and the later interviews did not identify any additional codes or diversely different experiences or views. Data saturation was reached after seventeen interviews [19].

We conducted overt participant observations to investigate the functionality of the ePPR. Each observation was conducted by a review of the ePPR function under the guidance of an ambulance professional. Observations were conducted individually by one researcher who also conducted the interviews. A template for writing field notes was established with the sections: participants, ePPR, and the three themes from the interview guide (perceived benefits, perceived barriers, and perceived influencing factors). Please see S2 File for the template. We recorded field notes during and immediately after the data collection to improve the validity of the data. Subsequently, the field notes were transferred into digital observation protocol and entered into NVivo for analysis.

#### Data analysis

All qualitative data were analysed using a hermeneutic phenomenological approach. The hermeneutic phenomenological analysis was conducted in a circular process since the understanding of the data became enriched from the numerous readings of the data [7]. Interviews and observations were analysed using thematic analysis, a qualitative descriptive approach for identifying, analysing, and reporting themes within data [20]. The thematic analysis was based on the approach described by Clarke and Braun, which includes six phases: Becoming familiar with the data, generating initial codes; searching for themes; reviewing themes; defining themes; and producing the report [20]. Data from the interviews and the observations were analysed and discussed jointly by two authors (JK,LM) to ensure the rigour of interpretation. We used Clarke and Braun’s 15-Point Checklist of Criteria for Good Thematic Analysis [20]. The analysis was performed using NVivo software.

*Ethics approval and consent to participate*. Permits have been obtained from the Region of Southern Denmark for data processing and disclosure of patient record information (Journal nos. 21/14737 and 21/9883). The data processing and data storage was performed on a secure server. By Danish legislation, interviews and quality studies do not require approval from the Committee on Health Research Ethics. All interviewed ambulance professionals signed written informed consent. The right to be anonymous and to withdraw from the study at any given time was highly emphasised.

## Results

### Phase one: The quantitative analysis

From 2016–2020, 385,165 ambulances were dispatched following a call to the emergency dispatch centre.

#### Data completeness

**Fig 3:**
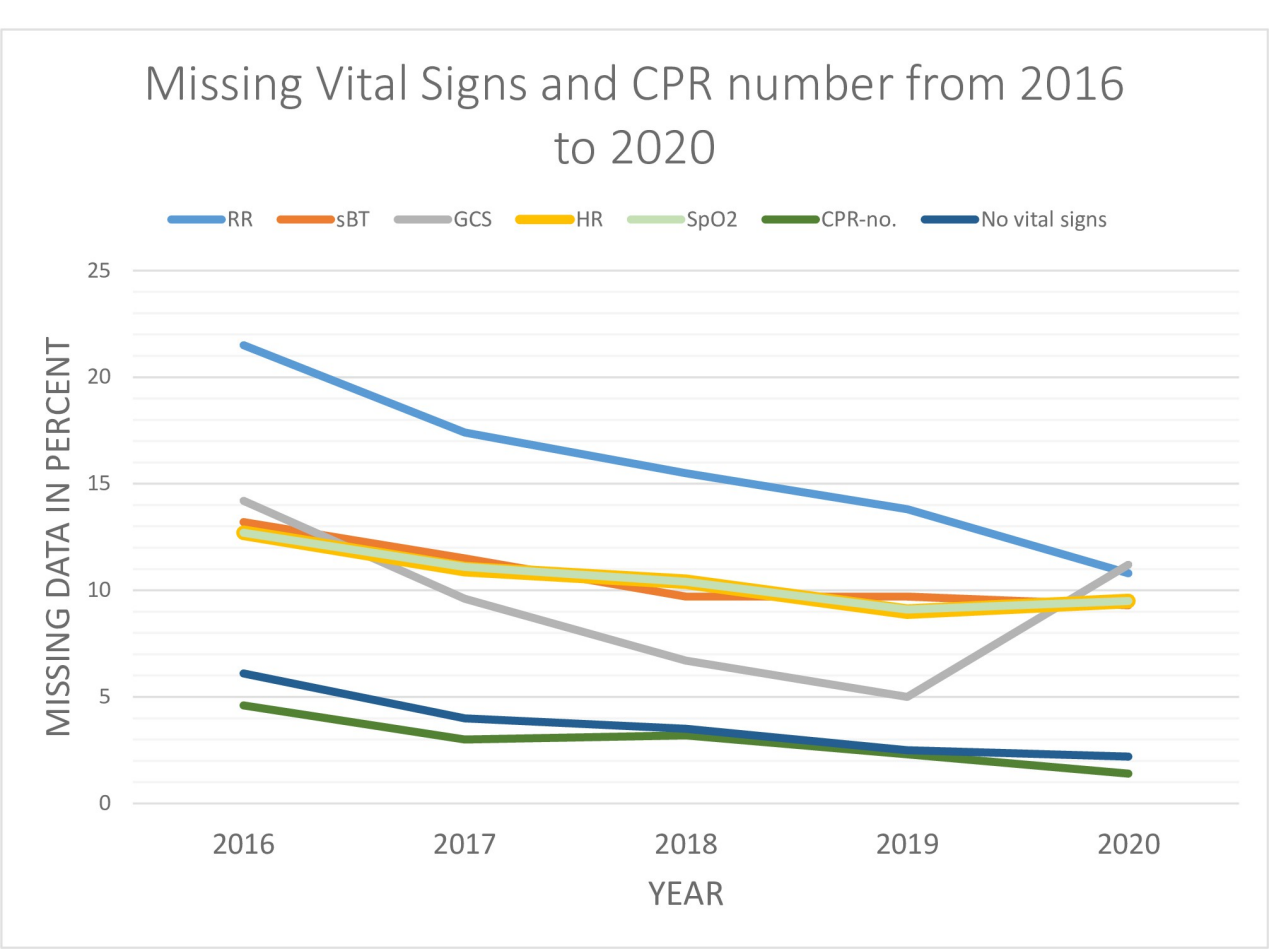
The proportion of patients without registration of vital signs from 2016 to 2020.

**Fig 4:**
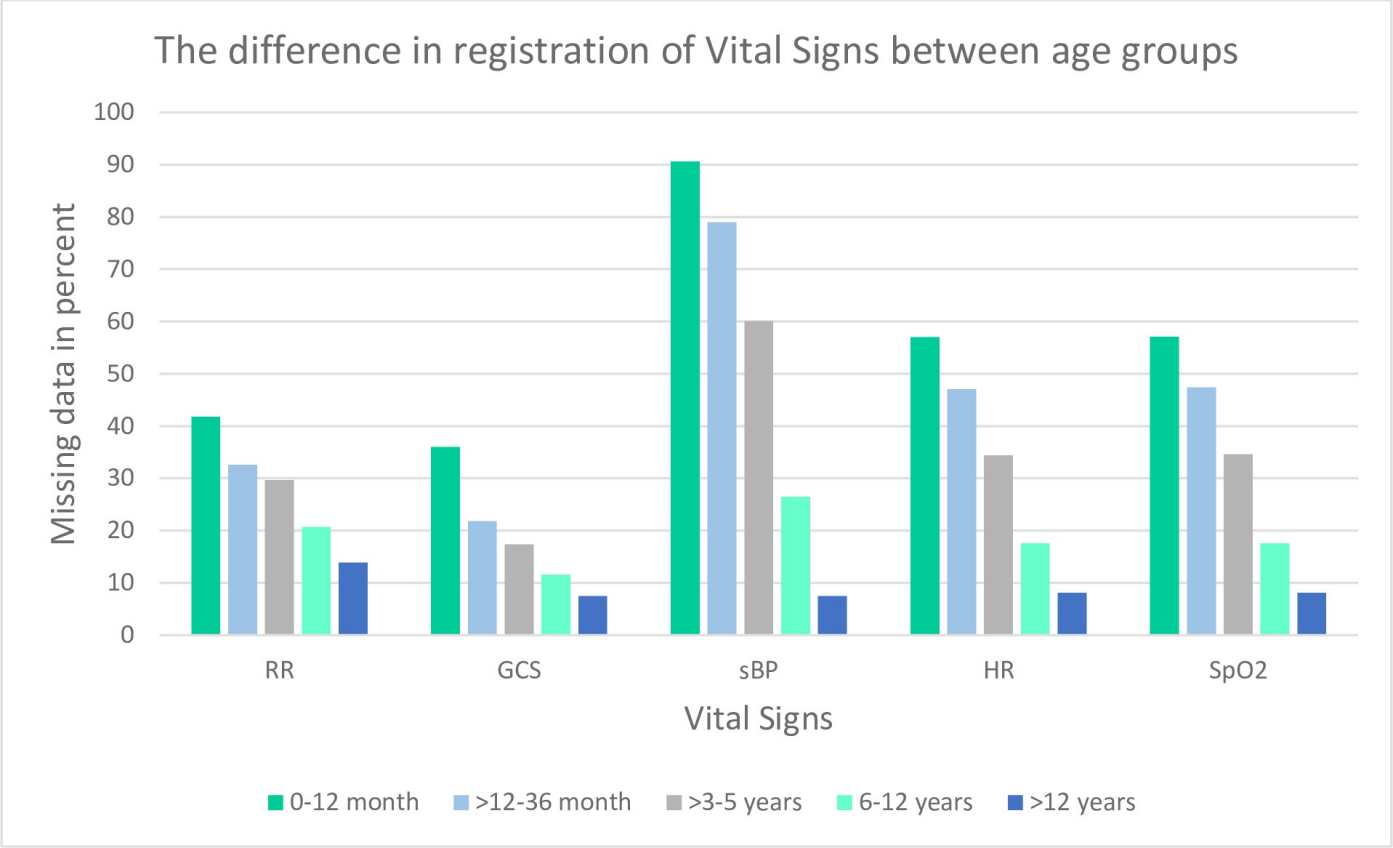
The difference between age groups shown in percentages of patients with missing vital signs.

Overall, the amount of missing vital signs and CPR numbers in the ePPR registrations decreased from 2016 to 2020. However, the proportion of missing registration of GCS, HR, and SpO_2_ increased from 2019 to 2020. From 2019 to 2020, GCS increased from 5% to 11.2%, HR increased from 9% to 9.5% and SpO2 from 9.1% to 9.5%, respectively. In general, the proportion of patients with missing registration of HR was largely similar to the proportion of patients with missing registration of SpO2. Missing CPR numbers increased from 3% in 2017 to 3.2% in 2018. In 2020, 11.2% and 10.8% of all included patients had missing GCS and RR registrations. The number of patients with no vital signs decreased by more than half from 6.1% in 2016 to 2020 2.2% in (Fig 3). When stratified by age groups, children <12 years had the highest proportion of missing vital sign registrations (Fig 4).

According to the local internal guidelines for prehospital providers, all non-identifiable patients should be registered with an interim identification bracelet number. However, in 2020, 23.5% of the patients without a registered CPR number did not receive an identification bracelet.

We also investigated the completeness of the initial triage level of the patients. In 2016 and 2017, two-thirds of the patients did not have their triage level registered. In 2019 and 2020, this level of missing values was reduced to between one-thirds and a quarter of the patients.

#### Data correctness

According to our cut-points for the vital signs, 0.01% of the RR were impossible values. There were no impossible values within the remaining vital signs. Overall, the data correctness for CPR numbers was improved from 2016 to 2020.

In 2020, only 24.2% of the registered identification bracelet number included the obligatory eight digits.

### Phase two: The qualitative analysis

In phase two, we interviewed seventeen ambulance professionals at four ambulance stations. The median age of the ambulance professionals was 41 years and the median length of service was 15 years. The characteristics of the participants are shown in Table 1.

**Table 1:** Demographics of the participants from phase two.

Variable		N
Gender	Male	13
	Female	4
Age	30–40	8
	41–50	6
	51–60	3
Education	EMT student	2
	EMT	10
	Paramedic	5
Years of experience	0–10	5
	11–20	7
	21–30	4
	>30	1

**Fig 5:**
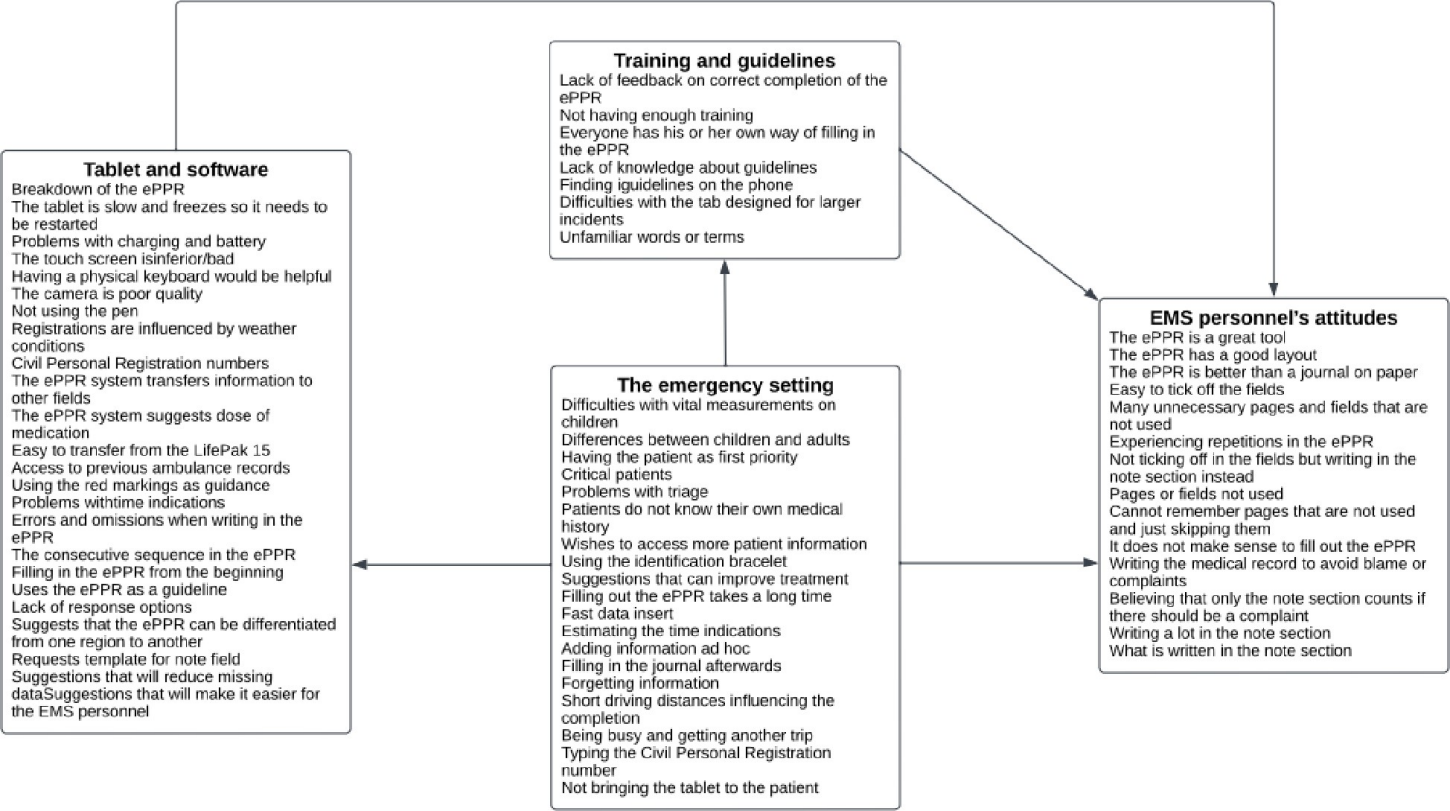
The first thematic map.

**Fig 6:**
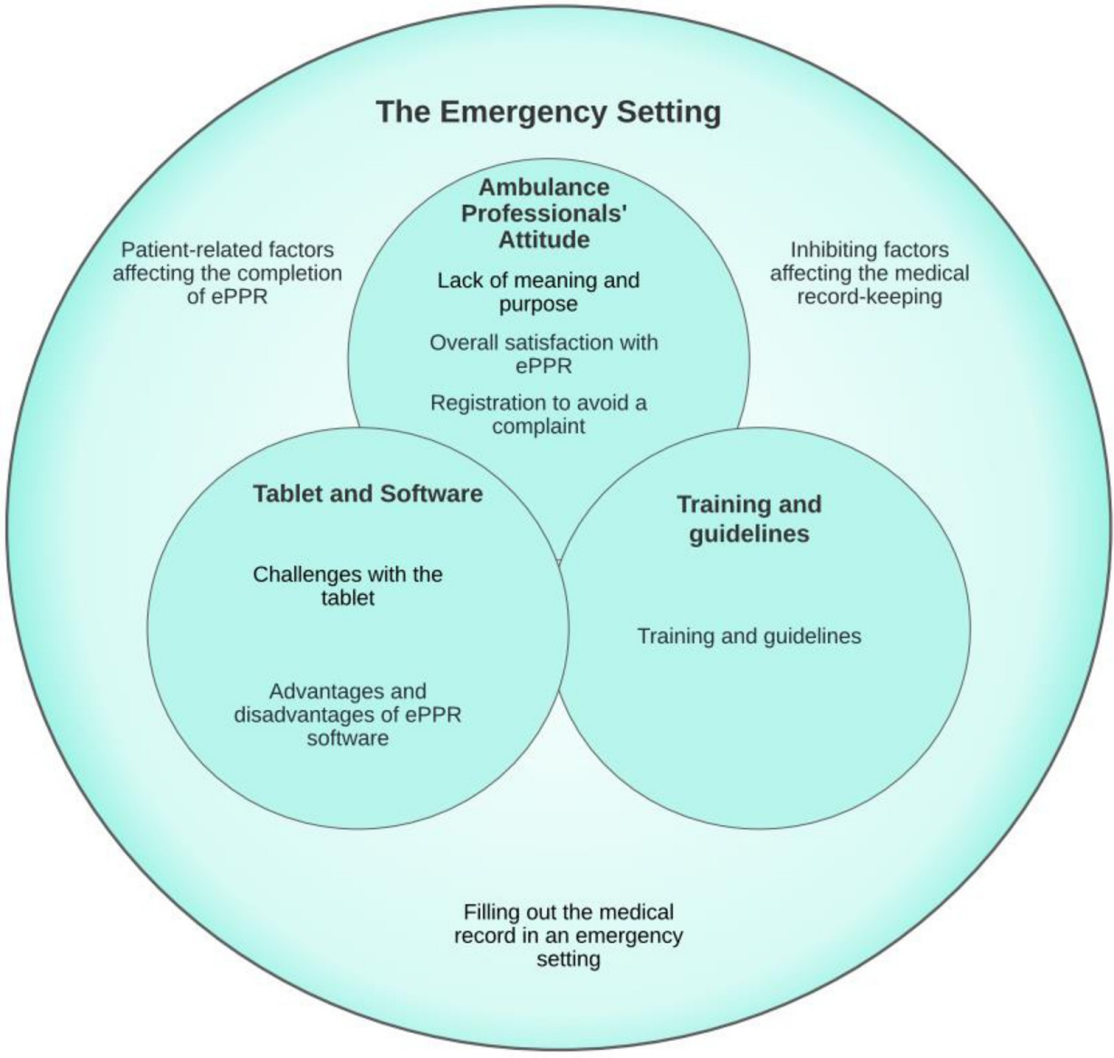
The final thematic map.

We identified four themes and nine subthemes during the iterative thematic analysis. The main themes were the ambulance professionals’ attitudes, the emergency setting, training and guidelines, and the computer tablet and software. The codes for each theme are shown in Fig 5. The themes and subthemes are shown in Fig 6.

#### Ambulance professionals’ attitudes

*Overall satisfaction with ePPR*. Overall, the ambulance professionals described the ePPR as an essential work tool and highlighted a well-structured ePPR layout and the usability of tick-off boxes that in many cases rendered text unnecessary. The ambulance professionals often compared the ePPR to the alternative, the paper record, when expressing positivity. For instance, an ambulance professional expressed,

”*Generally*, *I am happy about the ePPR instead of paper”*.

”You are only made aware of how good it [ePPR] is on the few occasions when it’s out of service and we have to use the paper records”.

*Lack of meaning and purpose*. Despite a general satisfaction when comparing the ePPR with paper records, the participants mentioned several factors that influenced the documentation. Most of the ambulance professionals reported that ePPR included many unnecessary sections, which caused frustration. One said,

“*We don’t use that many of all the tabs*. *It could probably all fit on one tab… there is a lot*. *Really a lot of fields that you rarely use”*.

As an example of the tabs not used, ambulance professionals mentioned a triage field “new triage”, which is no longer in use, and old research projects. Several of the ambulance professionals could not recall the tabs they did not use and described just skipping them. Despite the skipping of unnecessary tabs, 11 of the ambulance professionals felt like they were repeating themselves when filling in the ePPR:

”*We register lots of things twice… It is wasted time”*.

”*It is pleonasm*. *It seems silly*. *We are time pressured enough as it is”*.

In contrast, two of the ambulance professionals mentioned not ticking off the boxes but writing the information in the free-text section instead. One of them said,

”*I don’t want to have to repeat myself all the time… Then it just goes in the free text field”*.

Some felt it was pointless to complete the medical record because the information would not be of importance to the patient or used by the in-hospital personnel:

”*What is the point of me filling this in*, *when we arrive [at the emergency room]*, *and it does not affect the patient’s course*, *you know”*.

In addition, one ambulance professional described the pointlessness of prehospital triaging in the ePPR because the ambulance personnel often have to complete the triage after the patient has been handed over in the emergency room.

*Registration to avoid complaints*. Several ambulance professionals felt the purpose of completing the medical records was to avoid complaints.

”*… they will be down on you*, *if you… You will be tried at the Patients Complaints Board and all sorts*, *right*. *So you have to document everything*, *so it’s in order”*.

”*I will write exactly what I need to survive”*.

A few ambulance professionals mentioned they had been informed that the only thing legally valid was the written information in the free-text section in case of complaints. Several pointed out that they wrote a lot in the free-text section to be sure it was adequate, believing that longer descriptions in the free-text field would provide better insight into the patient.

”*It may be a lot of text*, *but I believe it is necessary because… You can’t see the same in the tick-off boxes”*.

*The emergency setting*. Ambulance professionals described the completion of medical records to be affected by the working conditions in a pre-hospital setting.

*Inhibiting factors affecting the medical record-keeping*. The majority of the ambulance professionals described not bringing the ePPR tablet with them to the scene of injury but only using it in the ambulance. They reasoned this with the fact that the ePPR cannot save the patient’s life and that they have a lot to carry already. The ambulance professionals mentioned not having much time to fill in the record in cases with short driving distances from the prehospital scene to the hospital. Therefore, some described choosing to stay at the scene to perform the first measurements of vital signs to be able to fill in the most important part of the medical record before arriving at the hospital:

”*Well*, *let’s just sit out here for a bit*, *or else we’ll arrive at the hospital after 10 minutes and we won’t have anything… we have to have something to work with”*.

The ambulance professionals described having the opportunity to fill in the medical record at the hospital after handing over the patient but also highlighted that it could lead to missing patient information. Several ambulance professionals mentioned that it could be difficult to remember what happened especially if they got another run before they finished filling in the record from the previous run.

”*But then there is the problem of not remembering exactly what happened”*.

”*Well there is always the element of you filling in the medical record and then you’re called and you have a new run… Then it starts to get a bit problematic*. *Because you’re in a mindset where you have to remember the run you’ve just had”*.

*Filling out the medical record in an emergency setting*. Even though the ambulance professionals overall found entering information into ePPR quick, several mentioned the increasing levels of the required information to be time-consuming:

”*The medical records are getting longer and longer*, *you know*. *We write more and more and document more and more”*.

Because of the work environment with time pressure and often short patient contacts, several participants mentioned that they often estimated time indications in the ePPR, while one participant noted the advantage of recording information continuously:

”I can record the information I receive or the medications I give continuously. Then I don’t have to make sense of it afterwards”.

*Patient-related factors affecting the completion of PPJ*. Several factors concerning the patients affected the documentation and registration. These were factors not relating to the documentation itself but more to the surrounding factors influencing documentation. If the patient was in a critical condition, ambulance professionals often filled in the majority of the information afterwards. The ambulance professionals expressed that the patient care and treatment had first priority and the registration in the medical record system to come second.

”*Of course*, *it’s important that you register*, *but it’s always more important that the patient survives”*.

Entering data into the medical record could also be challenging if the patient was a child. This was often a consequence of difficulties with obtaining the vital signs, and not entirely with the documentation itself. The difficulties with measuring vital signs in children often led to a more clinically related assessment of the patient, which had to be explained in the free-text section and not the vital signs tab in the ePPR:

” *I often use my notes in those instances*, *because I’ll write my way out of it*. *What I see*. *Because sometimes it’s difficult*, *and they won’t corporate and it all escalates”*.

”*Then it ends in the free text field*. *Yeah*, *exactly*. *We all know that the SpO2 monitor on the [defibrillator] isn’t that useful on a child”*.

In addition, the ambulance professionals did not find GCS useful for children and described the layout in the ePPR as not optimal to use when caring for children.

”*In my opinion*, *ePPR is difficult to use regarding children”*.

At times, the ambulance professionals found it difficult to obtain information on the patients’ medical history and several described that patients often do not know their medical history. Consequently, they had difficulties entering information on previous diseases and medication into the medical record.

”*Are you ill*? *No*. *Are you taking any pills*? *Yes*. *Well*, *then you’re probably ill”*.

Therefore, many ambulance professionals wished to have access to more patient information. This was both to save time but also to improve the treatment. One said,

”*You know*, *for me*, *it’s all about the information we can retrieve about the patient*. *Because a lot of important information is lost*, *and we spend lots of time trying to get the information other places”*

When touching upon improvements in the treatment, a few ambulance professionals thought it would be beneficial to see the current electrocardiogram (ECG) and previous ECGs in the ePPR to compare and enlarge the ECG and to avoid the paper. In addition, they requested the fields with indication, contraindication, dosage suggestions, side effects, etc. to be auto-completed to guide the ambulance professionals when they use the medicine.

The pre-hospital identification bracelet, which ambulance professionals use for non-identifiable patients, was also described as a challenge at times. Even though the majority of the ambulance professionals were familiar with the guidelines for the identification bracelet, they described problems with the bracelet scanner and errors in the previous bracelets. Therefore, they entered the bracelet number manually instead of scanning the number in contrast to the local internal guideline recommendation.

*Training and guidelines*. All ambulance professionals mentioned training and guidelines in relation to documentation. Despite some training opportunities, the majority of the ambulance professionals expressed a notion of not having received any training in filling in the ePPR. One of the ambulance professionals emphasized:

”*I had no training when I started using ePPR… In 2015*, *we got it in the car*, *and that was just it”*.

Still, some ambulance professionals expressed they could handle the job easily referring mainly to the technical aspect of the ePPR and not the content. When one of the ambulance professionals was asked if he felt he could manage the ePPR despite not having sufficient training, he replied:

“*Well*, *I belong to a generation who have grown up using computers*, *right… So that’s right up my alley”*.

Several ambulance professionals expressed receiving no feedback or audit on the correct completion of the records, and as a result, each had their way of filling in the ePPR. Furthermore, they mentioned having to train the apprentices and passing on their own ways.

“*I don’t have my records scrutinized*. *So my guess is that I have my way of doing it… I am not sure that it’s done right at all”*.

Because of a notion of insufficient training in the use of the ePPR, the ambulance professionals were confused about some tick-off boxes and unsure about what some fields involved. Many expressed that they became unsure of how to fill in the tab designed for larger incidents when they were under pressure. From several ambulance professionals’ point of view, this was the only tab they had received training in using, but because of the rare use of it, most had forgotten exactly how to fill it in.

#### Tablet and software

*Challenges with the tablet*. Factors associated with the tablet and the software affected the ambulance professional’s completion of the records. One thing the ambulance professionals expressed annoyance about was breakdowns of the ePPR and having to fill in a paper record instead. However, more often, the tablet was slow and froze or locked up and needed to be restarted. In general, ambulance professionals described problems with the hardware such as poor battery capacity, difficulty with placing the tablet properly in the chargers, poor quality of the inbuilt camera, and a faulty touch screen. The ambulance professionals often experienced that the touch screen did not register the touch properly making it difficult for the ambulance professionals to enter data or prose into the medical record. This caused the ambulance professionals to shorten the records.

”*You may have to write a little less than you normally would*, *because it’s a frustration that you have to go back and correct the mistakes you make all the time”*.

Despite the challenges with the touch screen, no ambulance professionals described using the touch pen mounted on the side of the tablet because they found this challenging. However, the ambulance professionals believed they would write a more adequate record if they had a physical keyboard.

*Advantages and disadvantages of ePPR software*. There are also substantial advantages to an EHR. The ambulance professionals described red markings appearing at tabs requiring essential or compulsory information as a guidance believing that the record is finished when all the red markings are filled in.

“*And then I like that thing with the red in the places where you’re missing something*. *Because sometimes you just forget something… And you don’t do that anymore*. *Not in the same way as before anyways”*.

In the same way, they mentioned using the ePPR to remember to ask the patient about all the relevant information and to carry out the required examinations of the patient. The consecutive sequence of the tabs influenced which questions and examinations they would prioritise.

”*That questioning technique we use when we’re talking with the patients is the same one integrated into the ePPR*. *In that way it’s assisting us in remembering everything*, *you know”*.

The EHR allowed the ambulance professionals to access the patient’s previous ambulance records, which the ambulance professionals often used. The ambulance professionals highlighted the possibility of transferring the measurements from the vital signs monitor to the ePPR as an advantage. It was described as time-saving and ensured that as many measurements as possible were recorded in the ePPR. Other functions that helped the ambulance professionals in their daily work were the dosage suggestions when they enter medications into ePPR and the automatic validation of the CPR numbers when entered.

On the other hand, there were also challenges associated with the tablet and software. The automatic time stamps could prevent the documentation of the correct time stamps. For example, the ambulance professionals described receiving an error message when attempting to document the onset of pain because the onset was before the ambulance arrived.

A large proportion of the ambulance professionals mentioned a lack of response options, for example under “previous illnesses” and under “reason for contact” and highlighted this as a disadvantage of the predetermined response options.

The ambulance professionals suggested several improvements such as a template for the free-text section with predetermined headings, an option to differentiate between medical records from different health regions, the possibility to create a medical record when the tablet is offline, and being able to transfer already entered information into the free text field.

## Discussion

In this study, we examined the data quality of the ePPR and explored possible reasons for our findings with semi-structured interviews. Overall, we found an improvement in data completeness and data correctness from 2016–2020 and a decline in the proportion of patients who had no measurements of vital signs. The largest degree of completeness was observed for the automatically measured vital signs: BP, HR, and SpO2. We found a small amount (0.01% of the RR) of clinically implausible vital signs. Overall, our findings on data completeness are supported by another registry-based study from 2020 [18]. In that study, the authors found that the completeness of the prehospital vital sign registration improved over the years with less than 10% having no vital signs measured [16]. Previous studies reported the poorest data completeness was found in registration of RR [5, 16, 21].

We explored barriers and facilitators to the data quality of ePPR data and the ambulance professional’s attitude to the use of the ePPR. The main themes included factors related to ambulance professionals’ attitudes, the emergency setting, the tablet and software, and training and guidelines. In accordance with our findings, Topas et al. described that barriers to completing medical records included time-consuming and slow systems, too many keystrokes to record simple information, multiple unnecessary sections that interrupt clinical thinking, and a lack of users’ training [2].

The ambulance professionals’ attitudes to the ePPR could be a barrier for data completeness and data correctness. The ambulance professionals experienced a lack of meaning and purpose in filling in the ePPR, making them less likely to complete the entire medical records. Strudwick et al. examined nurses’ perceptions of electronic health record use in an acute care hospital setting [22]. The nurses described challenges with their workload related to EHR use and identified added workload with multiple places to document the same information and the lack of clear organizational expectations for use of the EHR [22]. The participants in our study described similar challenges with the continuously added pages and documentation requirements affecting their attitudes related to ePPR use. You could imagine that this could be a barrier to high-quality patient care.

The ambulance professionals mentioned barriers associated with working in an emergency setting i.e. critical patients, short driving distance, being busy, and responding to another emergency run before information concerning the previous task had been entered into the ePPR. Another study reported that documentation had second priority and described difficulties with completing documentation in the ePPR before arriving at the hospital due to short driving distances [10]. This could affect data completeness and data correctness because the ambulance professionals described that it was commonplace not to be able to fill in the record while caring for the patient. Thus, they often completed the ePPR afterward. This leaves a risk of forgetting important patient information and may prevent data completeness and data correctness.

Our data showed that children <12 years accounted for the majority of the population with missing vital sign registrations (see Fig 4). The ambulance professionals experienced challenges obtaining the vital signs in children, and as a result, often focused on a clinical assessment of the child. This assessment, devoid of measureable vital signs, was frequently documented in the free-text section and not in the vital signs tab in the ePPR. Previous studies have found similar problems with not recording vital signs in children [23–25]. It can be difficult to measure blood pressure in children—for example, readings are likely to be falsely high in crying toddlers, and an appropriately sized cuff may not be available [26]. The existing literature emphasises the importance of measuring vital signs in children [25, 27]. A single set of vital signs undertaken before arrival at a hospital can identify the group of children who are at higher risk of an adverse in-hospital outcome [27]. In another study by Corfield et al., oxygen delivery, GCS, HR, and SpO2 together were deemed to be appropriate factors to discriminate between children (< 16 years) who died within 30 days or were admitted to the ICU within 48 hours of emergency ambulance transfer to hospital [25]. As such, lack of measurement and registration of vital signs in children is an area of improvement identified in this study. Future studies focusing specifically on prehospital vital sign measurements in children and addressing possible improving interventions are needed.

We identified additional challenges in the use of equipment. Despite the possibility of entering a patient´s CPR number electronically by scanning the citizen´s medical insurance card into the ePPR, the ambulance professionals mentioned that they often entered the CPR number manually, because of problems with the bracelet scanner. This may affect data correctness because of the risk of typing errors. This emphasises the importance of ambulance professionals being trained in the correct use of the equipment, as it is important to be able to identify the patient on arrival at the hospital to gain access to further patient information, which may be crucial to future treatment.

The ambulance professionals described problems with the hardware such as poor battery capacity, difficulty with placing the computer tablet properly in the chargers, poor quality of the camera, and a faulty touch screen. These are some fundamental factors that, implicitly, may affect data completeness and data correctness. These factors were considered an annoyance for the ambulance professionals and affected their attitudes towards the ePPR in a negative direction. If there is low battery life on the computer tablet, the ambulance professionals can only use the tablet when it is placed in the charger in the ambulance. Furthermore, the ambulance professionals expressed frustrations with the touch screen. The ambulance professionals believed they could write more elaborate medical records if they had a physical keyboard. Such a keyboard is, however, not available in all of the ambulances. This may affect the data quality of ePPR.

We observed that the consecutive sequence in entering data in the ePPR affected the completion of the medical record. The increased amount of missing registration of GCS from 2019 to 2020 may be caused by the field related to the registration of the GCS has been moved from one section of the computer tablet to another in 2020. Placing the pages and fields in a prioritised sequence could enhance data completeness as the majority of ambulance professionals expressed that they filled in the record ad hoc. Consequently, it may be important to keep this in mind when developing ePPR systems. The ambulance professionals described using the ePPR’s red markings (signaling mandatory requirements for filling out the ePPR) as a guide. However, these red markings do not necessarily correspond with the local internal guidelines for prehospital providers and thus may create a false perception of correct ePPR completion. It could be advantageous to use these red markings to achieve even better data quality by streamlining the design of the computer tablet with the local Danish guidelines.

Several ambulance professionals reported a notion of insufficient training in the completion of the ePPR and mentioned having to train the EMS students and passing on their own habits of entering data. The notion of insufficient training in filling in the ePPR could be a barrier for data completeness and data correctness. Not having enough training caused a lack of knowledge about the guidelines regarding the completion of the ePPR. Several ambulance professionals also expressed that they had not received any feedback or audit on the correct completion of the records, and as a result, each had their habit of filling in the ePPR. Poor and insufficient training and lack of technical/educational support for users create potential barriers to using EHR [6, 28–30] and may prevent a systematic and homogeneous approach to filling in the record. Participants from a Chinese study mentioned that it is important for health care workers to receive education and training regarding the utilization of EHR system functions and systematic documentation is the basic prerequisite to enhancing data quality [31].

The development and the implementation of EHR systems should focus on the needs and realities of the field in which they are put into practice. This requires the inclusion of healthcare providers’ satisfaction, “rather than only focusing on administrative and managerial functions, and financial performance” [32]. A study from Finland concluded that the development of EHR systems should consider the perspectives of the main user groups and the contexts of healthcare work [33]. Consequently, our results highlight barriers described by the users themselves, the ambulance professionals, which should be taken into account when developing and implementing ePPR system.

Overall, the findings in this study underline the importance of evaluating and including user feedback, as this is crucial to continuously improve data quality. Small changes to the existing system could perhaps substantially improve data quality. Our findings also point to several areas of improvement requiring more complex interventions, that in turn could improve the overall data quality. First, vital signs measurements on children are a challenging area. Future studies should focus on the development of interventions that could facilitate easier and correct measurements on children. Second, continuous training, perhaps as part of regular clinical training, may improve data quality. Training could facilitate systematic and homogenous completion of ePPR medical records. Additionally, audits of medical records and continuous monitoring the completion of medical records may be beneficial to identify challenging areas and addressing possible solutions. If these results are taken into account, it will be possible to enhance the data quality, which will improve the possibility of quality assurance of the prehospital treatment [3], the patient care [4, 34], and the quality of the scientific work [35], and thus the future patients.

### Study strengths and limitations

When used in combination, quantitative and qualitative methods complement each other and allow for more robust analysis, taking advantage of the strengths of each [36]. A major strength of the quantitative phase of the study was a long study period together with a large study population consisting of all emergency runs in a free-access healthcare system. This ensured that the population included all patient and age groups. The minimum of impossible values may be underestimated because we did not define any lower limits for the vital signs. However, this was a conscious choice to include critical patients with extremely low vital signs, for example, patients with cardiac arrest where vital signs may be zero, absent, or physiologically, extremely low. We only included the last registered measurement of each vital sign in the analysis, as we aimed to investigate data completeness and correctness. In addition, we only included selected sections from the ePPR. It may have given a broader picture of the data quality if we included all pages and fields from the ePPR. Previous researchers have identified five dimensions of data quality (completeness, correctness, concordance, plausibility, and currency) [15]. We only included data completeness and data correctness. If we had included all five dimensions, we may have found a broader perspective of the data quality. Future research could consider including all five dimensions of data quality and all sections from ePPR.

In our qualitative analysis, we included four ambulance stations in an urban area covering both a private and public ambulance service, but only from the Region of Southern Denmark. Thus, our study population may differ from ambulance professionals from other areas and thus may not be generalisable. In addition, the ambulance professionals in our sample volunteered to participate in the interview, so there could be selection bias. Nevertheless, it was a representative sample of the Danish ambulance professionals regarding age, gender, length of service, and level of education. A major strength of the qualitative study was that two authors with previous experience in qualitative research carried out the analysis. Sampling continued until saturation was reached and the later interviews not identify any additional. However, we did not, observe the ambulance professionals on scene in real-life situations. We might have identified further barriers and facilitators if we had observed the ambulance professionals on scene. It may be meaningful to include this perspective in future research.

## Conclusion

We found an overall high quality of data. However, some need for further improvements were identified as data registration was insufficient in some instances. There may be several reasons for insufficient data registration. The ambulance professionals’ attitudes to the ePPR, the mere fact that working in an emergency setting may be stressful, a notion of insufficient training in filling in the ePPR, and challenges associated with the computer tablet and software could be a barrier to data completeness and data correctness. Several of the themes and subthemes mutually influence each other. This should be taken into consideration when developing and implementing an ePPR system. It will also be advantageous to include the end-user and take into account that the system and computer tablet should be used in an emergency setting. The findings from this study will be relevant to include during the development of a new ePPR system or to improve the current ePPR system.

## Supporting information

S1 File Interview guide. (DOCX)

S2 File Observation guide. (DOCX)
