# Supplementary material for: The data quality and applicability of a Danish prehospital electronic health record: A mixed-methods study
Source: PLoS One. 2023 Oct 26;18(10):e0293577. doi: 10.1371/journal.pone.0293577 (PMC10602337; doi:10.1371/journal.pone.0293577)
Supplement: S1 File — (DOCX) [file pone.0293577.s001.docx]

**S1 Fig. Interview guide.**

| Place:  Date: | Start (time):  End (time): |
| --- | --- |
| **Briefing** | |
| Presentation of myself | I am from the Prehospital Reseach Unit, Region of Southern Denmark, and have a background as an emergency medical technician and a Master of Science in Public Health. |
| The study purpose | The purpose of this interview is to get your opinions on PPJ and hear about your experiences using PPJ. |
| Time frame | I expect the interview to last about 20 to 30 minutes. |
| Anonymisation | Your answers will be anonymous and handled confidentially. |
| Information to the participant | Participant is voluntary and you can always withdraw your consent. Feel free to ask questions along the way. You do not have to answer any questions you are not comfortable with. |
| Audio recording | Do I have your permission to audio record the interview? |
| **Questions** | |
| **Information on the participant** | Name: |
|  | Age: |
|  | Gender: |
|  | Educational level: |
| **Opening questions** | How many years have you been an EMT? |
|  | How long have you been an EMT at this specific ambulance base? |
| **Essential questions** | |
| **Themes** | **Questions** |
| Perceived benefits/ Perceived barriers | What do you like about PPJ? |
| Perceived benefits | What do you think works well in PPJ? |
| Perceived barriers | What does not work about PPJ? |
| Perceived barriers/ Perceived benefits / Perceived influencing factors | Is there something in your work day that influences your use of PPJ? *Both positively or negatively. If yes, what, and could you please describe further*  Is there something about PPJ that influences how you document in the medical record? *Both positively or negatively. If yes, what, and could you please describe further* |
| Perceived barriers | Do you have any suggestions for improvements to the PPJ? *If yes, which?* |
| Perceived influencing factors | What training have you received about PPJ? Do you think it was sufficient? |
| Clarifying questions | Is there fields or functions in the PPJ you rarely use? *You can look in the PPJ if you prefer.*  *If yes, which and why?* |
| Clarifying questions | Which fields in the PPJ do you use every time? *You can show me in PPJ if you prefer* |
| Clarifying questions | How do you use the free text field? What information do you note in the field? Do you write a lot or a little? |
| Clarifying questions | Does you documentation in the PPJ vary depending on the patient age (child vs adult)? *If yes, how and why?* |
| Clarifying questions | In what situations do you use PPJ bracelets? |
| Perceived barriers/ Perceived benefits / Perceived influencing factors | Do you have any recommendations if one were to make a new PPJ system? |
| **Closing questions** | Do you have anything to add? |
|  | Do you have any questions? |
