# Supplementary material for: The data quality and applicability of a Danish prehospital electronic health record: A mixed-methods study
Source: PLoS One. 2023 Oct 26;18(10):e0293577. doi: 10.1371/journal.pone.0293577 (PMC10602337; doi:10.1371/journal.pone.0293577)
Supplement: S2 File — (DOCX) [file pone.0293577.s002.docx]

**S2 Fig. Observation guide.**

| **Observation** | |
| --- | --- |
| Location:  Date: | Start (time):  End (time): |
| **Participants** | |
| Name:  Age: | Gender:  Educational level: |
| **ePPR** | |
| Comments on the functionality or use of ePPR: | |
| **Themes** | |
| Perceived benefits: | |
| Perceived barriers: | |
| Perceived influencing factors: | |
